# The use of anti stretch marks’ products by women in pregnancy: a descriptive, cross-sectional survey

**DOI:** 10.1186/s12884-016-1075-9

**Published:** 2016-09-21

**Authors:** Miriam Brennan, Mike Clarke, Declan Devane

**Affiliations:** 1 School of Nursing & Midwifery, Aras Moyola, National University of Ireland Galway, Galway, Ireland; 2 Centre for Public Health, School of Medicine, Dentistry and Biomedical Sciences, Institute of Clinical Sciences, Block B, Queen’s University Belfast, Royal Hospital, Grosvenor Road, Belfast, BT12 6BA Northern Ireland

**Keywords:** Pregnancy, Striae gravidarum, Topical products

## Abstract

**Background:**

Stretch marks (Striae gravidarum) are a cutaneous change occurring commonly during pregnancy. A variety of products are available and promoted as ways to prevent or reduce their development, but it is not clear what products are used most commonly. The objective of this study was to identify topical products used during pregnancy to prevent or reduce the development of striae gravidarum. We also explored issues around application of the product, cost incurred and influences on women’s decisions to use a product.

**Methods:**

In this cross sectional, descriptive survey we collected data from 773 women, via a paper (*n* = 707) or online (*n* = 66) questionnaire. Due to missing data in the online survey, 753 women at 36 weeks gestation or more were included in the analyses. Descriptive and inferential statistical analyses were undertaken.

**Results:**

Most respondents (*n* = 589, 78.2 %) indicated that they used a product to prevent or reduce the development of stretch marks during their current pregnancy. A large range of products were used and more than one third of women (*n* = 210, 36.5 %) had used two or more products. Bio-oil was the most frequently used product (*n* = 351, 60.9 %) and it was also the most frequently used product among women who used only one product (*n* = 189, 32.8 %).

**Conclusions:**

Many women apply one of the many products available to prevent or reduce the development of striae gravidarum. Bio-oil was the most commonly used product identified in this study. There is a need for high-quality evidence on the effectiveness of Bio-oil and other products.

## Background

Striae gravidarum, or stretch marks of pregnancy, are a common cutaneous physiological change occurring during pregnancy [1]. They are considered the most common connective tissue change of pregnancy [2] and affect both primiparae and multiparae women. Women of all racial groups are at risk [3]. Rates of occurrence vary [4], with reported rates ranging between 50 and 90 % [5]. Striae gravidarum usually first appear around the sixth and seventh month of pregnancy [6] but have being reported prior to 24 weeks gestation [7]. They occur most commonly during a first pregnancy but have been known to occur for the first time in a second pregnancy [4]. Striae vary in quantity and severity, frequently affecting the abdomen [8], breasts and thighs where there is greatest stretching of the skin [9].

Striae begin as 'reddish slightly depressed streaks’ [10], which lighten in colour over time [11], fading [12] to leave glistening [10], or pale wrinkled lines [13] on the skin by about 6 months after birth [14]. The exact aetiology of striae remains unclear [2, 5, 7, 8, 15–19]. Possible explanations centre on stress on the tissue or stretching of the skin and hormonal factors [2, 9]. At a micro level, it is suggested that changes occurring in the collagen elastin and fibrillin, which contribute to the tensile strength and elasticity of the skin [13, 20], are significant factors in their development.

While the cause of striae remains unclear, certain predisposing factors have been identified, albeit inconsistently. They include an inherent susceptibility to developing striae [13] or family history of striae [7, 16, 21, 22], higher maternal weight gain [5, 17, 22–24], younger aged mothers [5, 17, 21, 24–26], high pre-pregnancy body mass index [17, 22, 26] and a high infant birth weight [16, 17, 24]. Younger mothers are more likely to develop striae and to develop severe striae [17, 21]. More recently, geographic location and environmental factors [27] were found to influence the development of striae, while age was not found to be a predisposing factor [28].

Although striae are not considered a significant health issue, they can affect women in different ways and may cause distress to some women [4]. They may also cause pruritus [1, 7, 29] or discomfort [7] while some refer to them as ‘disfiguring’ [3, 7, 13] or as an aesthetic [17] or cosmetic concern [5, 22, 30, 31]. Some authors suggest that striae impact on women's perception of themselves and on their quality of life [4]. However, a cross sectional study on quality of life in Japanese pregnant women with striae [32] found that general quality of life scores did not differ between those with or without striae but that women with severe striae had significant higher emotion scores on the dermatology specific health related quality of life instrument (HRQoL) Skindex −29 [32].

Interventions for stretch marks include those that focus on prevention and those that focus on treatment [33]. During pregnancy, the focus is on prevention of striae or on reducing their severity and a wide range of products are available purporting to prevent or minimize the development of stretch marks. Consequently, women may use these products during pregnancy, many of which are considered cosmetic products [34] incurring significant expense [4]. However, the effectiveness of many products is unclear [35] due to the limited amount of research undertaken to date. A recent Cochrane Review [36] which included six trials involving a total of 800 women, found no high-quality evidence to support the use of any of the topical preparations identified in the review for the prevention of stretch marks during pregnancy. The authors recommended that preparations commonly used by women to prevent and treat stretch marks should be evaluated in large trials.

This cross sectional, descriptive survey, which is part of a planned investigation of topical products to prevent or reduce stretch marks in pregnancy, sought to identify the topical products used during pregnancy to prevent or reduce the development of striae gravidarum. We also explored issues around application of product, costs incurred and factors that influenced women's decision to use a product.

## Methods

We used a cross sectional, descriptive survey because of its suitability for ascertaining viewpoints [37] at one point in time [38]. Data were collected via a purposefully developed questionnaire in both paper (main study) and online format. The questionnaire contained 21 items chosen after an extensive search of the literature on stretch marks in pregnancy and discussions with researchers and clinical staff [38, 39]. The final instrument had both open and closed ended items, included 'skip logic', and mainly addressed behaviours [40] in relation to product application. Closed items required participants to tick one or more options from a choice of options [41]. The item seeking information on which product, if any, participants used asked participants to identify the product or products used by selecting 'all that apply' from a list of commonly used products generated from the literature and discussion with clinical staff. Response options included an option to select ‘other’ and add narrative to identify a product respondent may have used but which was not captured in listed response options. A similar item stem and response options were used for the item asking about information sources to help women decide which product to use (Questionnaire is available from MB).

Content validity testing, informed by the work of Lynn [42] and Polit et al. [43], was undertaken with the assistance of a panel of 12 experts. Criteria used for panel selection were mainly methodological and clinical expertise plus consumer representation. When tested, the instrument was found to have good content validity with each item having a content validity index value (I-CVI) ≥ 0.83 while the entire instrument had a content validity index of 0.94.

Data collection occurred over 16 months between July 2013 and April 2015. Data for the paper version were collected by one author (MB) and two research assistants, with support from staff in the antenatal clinic. Only one data collector was present at any time. Women were approached as they checked in or were waiting for their routine antenatal appointment at 36 weeks or more gestation. Almost all eligible women who were approached to participate agreed to do so. Women attending parentcraft sessions were recruited with the help of the parentcraft team. Completed questionnaires were deposited by women in a box on the clinic reception desk.

Potential participants were given information on the study including its purpose and what participation involved. Completion of the questionnaire was taken as an explicit indication of consent to participate in the study and this was outlined with the tenets of informed consent in the first section of the questionnaire. The online version of the questionnaire was supported by the online provider SurveyMonkey™ (https://www.surveymonkey.com/). This study was approved by the Clinical Research Ethics Committee for the Galway University Hospitals Group and by the Research Ethics Committee of the National University of Ireland Galway.

Participants were women who were at least 36 weeks pregnant attending the antenatal clinic and parentcraft education in a large regional hospital in the West of Ireland. Only English speaking women were eligible to participate, due to insufficient resources for translation of study material. The sample size for distribution of the main survey (paper questionnaire) was 692 women. This was calculated based on a total population size of 3500 (average births per annum in the study site) and 95 % confidence level, 5 % margin of error, which estimated a required sample size of 346. This was doubled, based on an assumption that the response rate would be 50 %. As the likely population size for the online survey was unknown, it was not possible to predetermine the sample size for this mode of data collection because women were notified through the maternity care advocacy groups and the number of women in these groups is unknown.

As data quality is contingent on respondents being able to understand what is being asked [39], pre testing and piloting of the questionnaire was essential. Colleagues assisted with this, specifically focusing on the interpretation and clarity of questions [39]. A pilot study was also undertaken to evaluate the questionnaire and the entire survey process [38, 44]. Respondents (*n* = 33) similar to the intended main sample completed the questionnaire and commented on the flow, length, ease of completion and acceptability [45]. No significant changes were required to the questionnaire in relation to layout or instructions following this preliminary testing.

Collected data were entered into SPSS version 21 manually [46] and checked and cleaned. Statistical analysis involved both descriptive and inferential statistics. Descriptive statistics included frequencies and measures of central tendency and variation while inferential statistics included Pearson Chi-square and Two proportion z test to explore relationships and differences in relation to product use between primigravida and multigravida women. Data are reported for completed items i.e. we did not impute missing values.

## Results

Of the 730 women asked to participate in the main survey, 707 agreed to do so, giving a response rate of 96.8 %. Of the 66 women who completed the online version 20 were ineligible because the woman’s gestation was not provided or she was under 36 weeks gestation. This left 46 completed online questionnaires and an overall total of 753 eligible participants (707 paper version, and 46 online).

The mean gestational age of respondents was 38 weeks (SD 1.5). First time mothers accounted for 40.2 % (*n* = 302) of respondents while the majority of respondents (*n* = 449, 59.8 %) were expecting their second or subsequent baby and the mean number of previous babies women had was 0.93. Most participants were Irish (*n* = 589, 78.3 %), followed by Polish (*n* = 58, 7.7 %), and 35 other nationalities were represented in the sample.

**Fig. 1:**
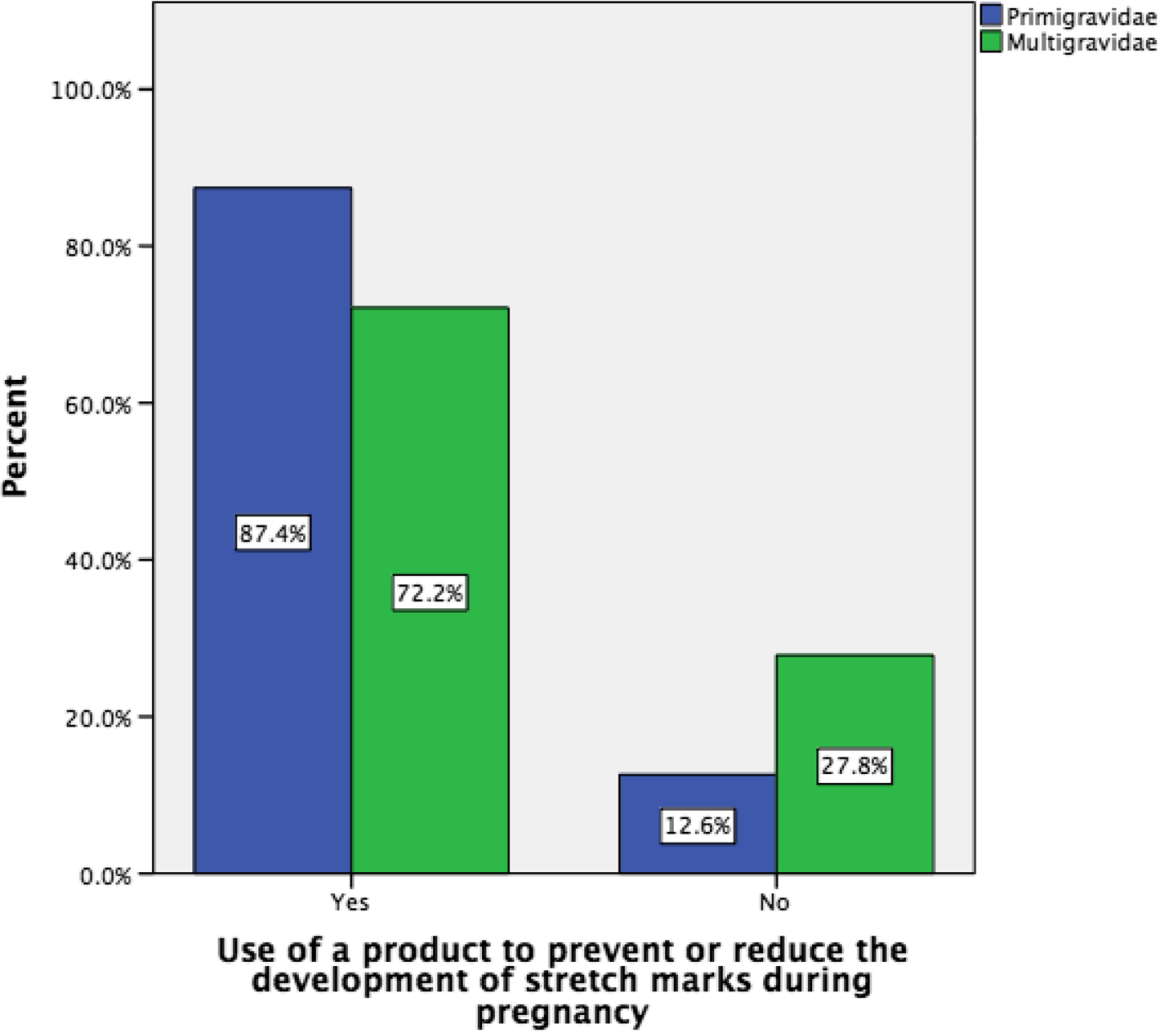
Use of a product to prevent or reduce the development of stretch marks by gravida

The majority of respondents (*n* = 589, 78.2 %) indicated that they had used a product to prevent or reduce the development of stretch marks during their current pregnancy. Of the women who used a product and completed the question on the use of specific skin products (*n * = 576, 98 %), 60.9 % (*n* = 351) used Bio-oil, followed by ‘other’ products (*n* = 202, 35.1 %), while the next most popular product was cocoa butter cream, which was used by 174 (30.2 %) women and cocoa butter lotion used by 50 (8.7 %) women (Table 1). A large range of products were included by women in the 'other' category with examples including baby oil (*n* = 31, 5.4 %), coconut oil (*n* = 16, 2.8 %) and almond oil products (*n* = 11, 1.9 %). Respondents also included some cocoa butter products (*n* = 4, 0.7 %) and many commercially available anti striae products in the 'other' category. Cocoa butter products (cream, lotion and other) were the second most popular product used (*n* = 228, 39.6 %). More than one-third of women (36.5 %, *n* = 210) used two or more products. When comparing primigravida and multigravida women, we found that significantly more primigravida women reported using a product compared to multigravida women (87.4 versus 72.2 %, *X*^*2*^ (1, *n* = 751) =24.7, p =0.000) (Fig. 1). However, there was no significant difference in the average number of products used between primigravida and multigravida (mean difference (MD) = 0.11, t (573) = 1.809, *p* = 0.071).

**Table 1:** Stretch mark products used

Product Name	Responses	Percent of overall respondents (*n* = 576)	Numbers (%) using specific product only	Numbers (%) using specific product plus one or more products
	*n*			
Bio-oil	351	60.9 %	189 (32.8 %)	162 (28.1 %)
Other product	202	35.1 %	97 (16.8 %)	105 (18.2 %)
-Baby oil	31	5.4 %		
-Coconut oil	16	2.8 %		
-Almond oil products	11	1.9 %		
-other	144	25 %		
Cocoa butter Cream	174	30.2 %	49 (8.5 %)	125 (21.7 %)
Cocoa butter lotion	50	8.7 %	10 (1.7 %)	40 (6.9 %)
Mama Mio tummy rub stretch mark oil	28	4.9 %	12 (2.1 %)	16 (2.8 %)
Olive oil	13	2.3 %	4 (0.7 %)	9 (1.6 %)
Revitol stretch mark cream	6	1.0 %	2 (0.3 %)	4 (0.7 %)
Germ oil	4	0.7 %	3 (0.5 %)	1 (0.2 %)

In relation to information sources that helped women to choose a product, 49.3 % (*n* = 278) of women based their decision on advice from friends, 23 % (*n* = 130) on product advertisement, 18.8 % (*n* = 106) on advice from a family member and 14.7 % (*n* = 83) on advice from the internet. In relation to health care professionals the pharmacist was the most frequently identified information source (*n* = 41, 7.3 %) followed by the general practitioner (GP) (3.4 %, *n* = 19) while midwives and obstetricians were consulted by 1.2 % (*n* = 7) and 0.2 % (*n* = 1) of women, respectively. Some women identified that they had used the product in a previous pregnancy or had got the product as a gift and therefore did not choose the product deliberately or incur any cost. Excluding the five (0.8 %) women who got the product as a gift, the average amount spent by women on products to prevent or reduce the development of stretch marks was €16–20 per woman. We found an association between the amount of money spent on skin products to prevent or reduce the development of stretch marks between primigravida and multigravida women. Significantly more multigravida women spent < €5 on skin products than primigravida (8.6 versus 3.8 % respectively, *p* = 0.015). However, at the upper spending range, significantly more primigravida women spent €51 or more when compared with multigravida woman (15.2 versus 9.2 % respectively, *p* = 0.029) (Table 2).

**Table 2:** Amount of money spent on skin products by gravida

Amount of money	Number (%) of Primigravidae (*n* = 263)	Number (%) of Multigravidae (*n* = 315)	*P*-value
< €5	10 (3.8 %)	27 (8.6 %)	*0.015
€5–10	38 (14.4 %)	62 (19.7 %)	0.122
€11–15	35 (13.3 %)	37 (11.7 %)	0.614
€16–20	45 (17.1 %)	57 (18.1 %)	0.827
€21–30	42 (16.0 %)	46 (14.6 %)	0.727
€31–40	27 (10.3 %)	36 (11.4 %)	0.689
€41–50	26 (9.9 %)	21 (6.7 %)	0.171
€ ≥ 51	40 (15.2 %)	29 (9.2 %)	*0.029

* *p* < 0.05

**Fig. 2:**
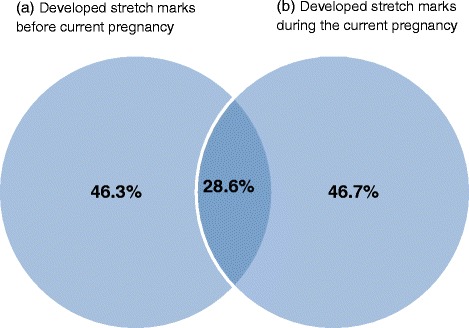
Percentage of women developing stretch marks both before and during current pregnancy Venn diagram depicts (**a**) % of women who developed stretch marks prior to current pregnancy (46.3 %) and (**b**) % of women who developed stretch marks during the current pregnancy (46.7 %). Intersection represents the % of women who developed stretch marks both before and during the current pregnancy (28.6 %)

**Fig. 3:**
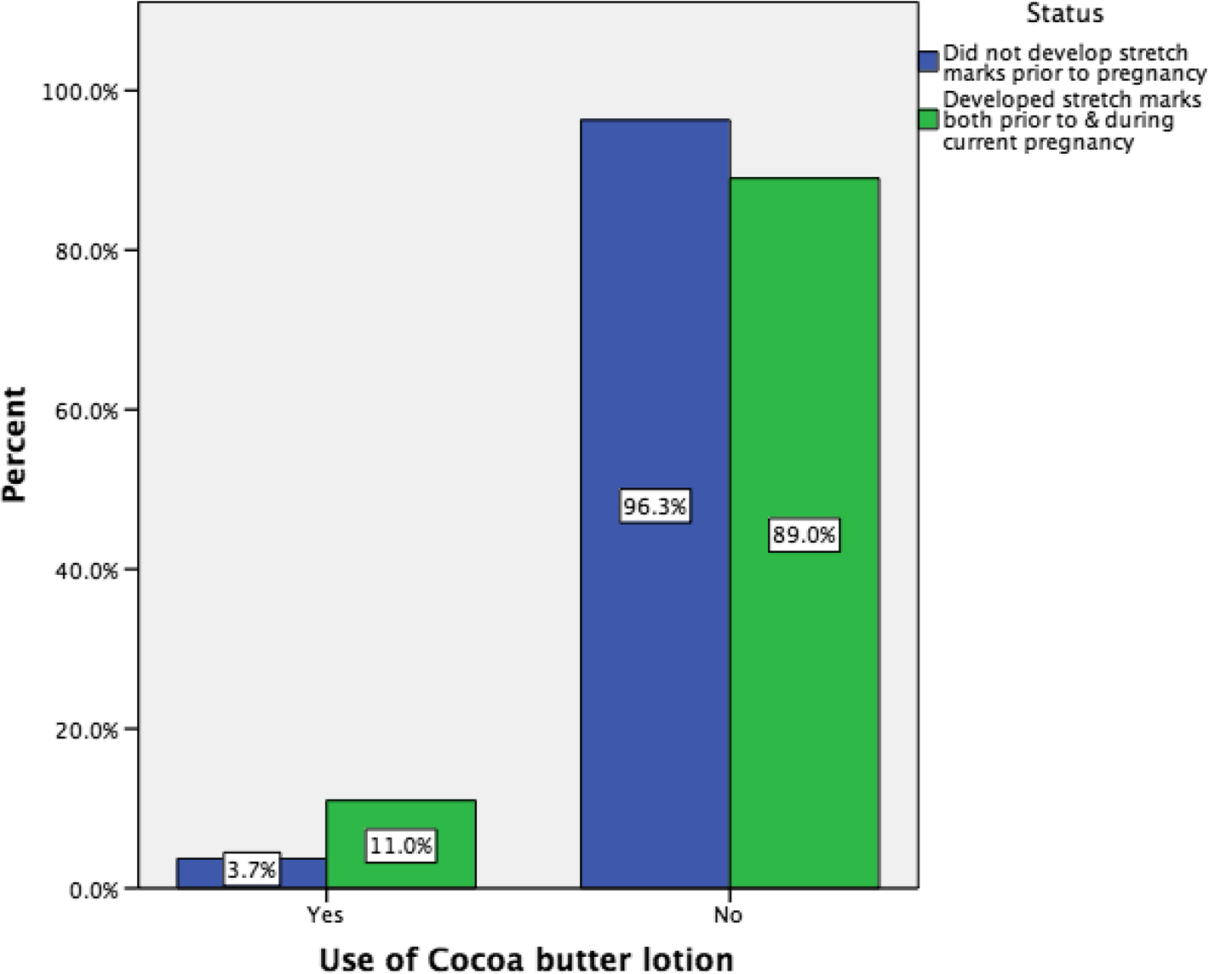
Use of cocoa butter lotion and timing of development of stretch marks

**Fig. 4:**
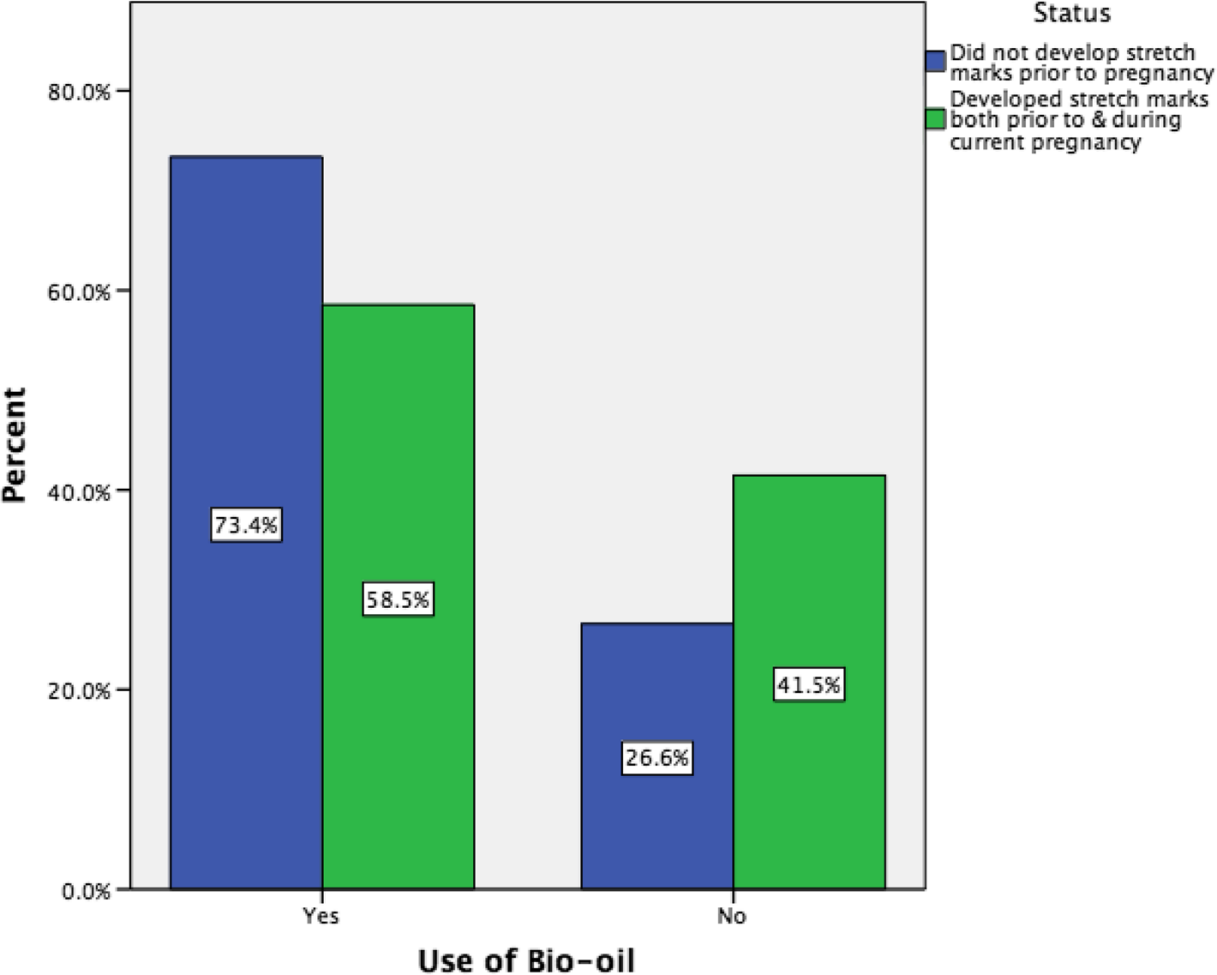
Use of bio-oil and timing of development of stretch marks

In this survey, 46.3 % (*n* = 342) of women had developed stretch marks before their current pregnancy and 46.7 % (*n* = 344) developed them during the current pregnancy. Of all respondents, 209 (28.6 %) developed stretch marks both before and during this current pregnancy (Fig. 2). On comparing primigravida and multigravida women, 48.1 % (*n* = 142) of primigravida and 45.9 % (*n* = 202) of multigravida women developed stretch marks during the current pregnancy. The majority of women (67.1 %, *n* = 232) classified the amount they got during this pregnancy as ‘a few’. A Chi-square test for independence indicated no significant association between application of a skin product to prevent the development of stretch marks and the development of stretch marks during this pregnancy (*X*^2^ (1, *n* = 737) = 2.174, *p* = 0.140), or with having developed stretch marks prior to the pregnancy, *X*^2^ (1, *n* = 739) =3.179, *p* = 0.075. When comparing women who developed stretch marks prior to and during the current pregnancy versus those who did not develop stretch marks prior to pregnancy but developed them during the current pregnancy, we found that women who developed stretch marks both prior to pregnancy and during the current pregnancy were significantly more likely to use cocoa butter lotion than those who did not develop stretch marks prior to pregnancy but developed them during the current pregnancy (11.0 versus 3.7 % respectively, *p* = 0.016) (Fig. 3). In relation to Bio-oil, we found those women without stretch marks prior to pregnancy and who developed them during the current pregnancy were significantly more likely to use Bio-oil than women with stretch marks prior to and during the current pregnancy (73.4 versus 58.5 % respectively, *p* = 0.009) (Fig. 4). There was no difference in relation to the other products like cocoa butter cream or olive oil.

Women were asked about the amount of time they spent applying the product and how often they applied it. Almost half of respondents (*n* = 262, 46.2 %) who used a product and completed this question (*n* = 567) had started to apply it in the first trimester and 266 (46.6 %) were applying it 7 days a week. The majority of women (*n* = 398, 71.6 %) applied the product once a day (women selected from a list of time options) and the mean time spent applying it was 3.8 min (range: 29.9, 0.1 to 30 min; median: 2.5 min). There was no significant association between the stage of pregnancy women were at (≤20 weeks or > 20 weeks) when they started to apply the product and being a primigravida or a multigravida woman (*X*^2^ (1, *n* = 566) =0.944, *p* = 0.331) nor was there a significant difference between the average length of time spent per day applying the product and being a primigravida or a multigravida woman (MD = 0.459, t (558) = 1.52, *p* = 0.129).

**Fig. 5:**
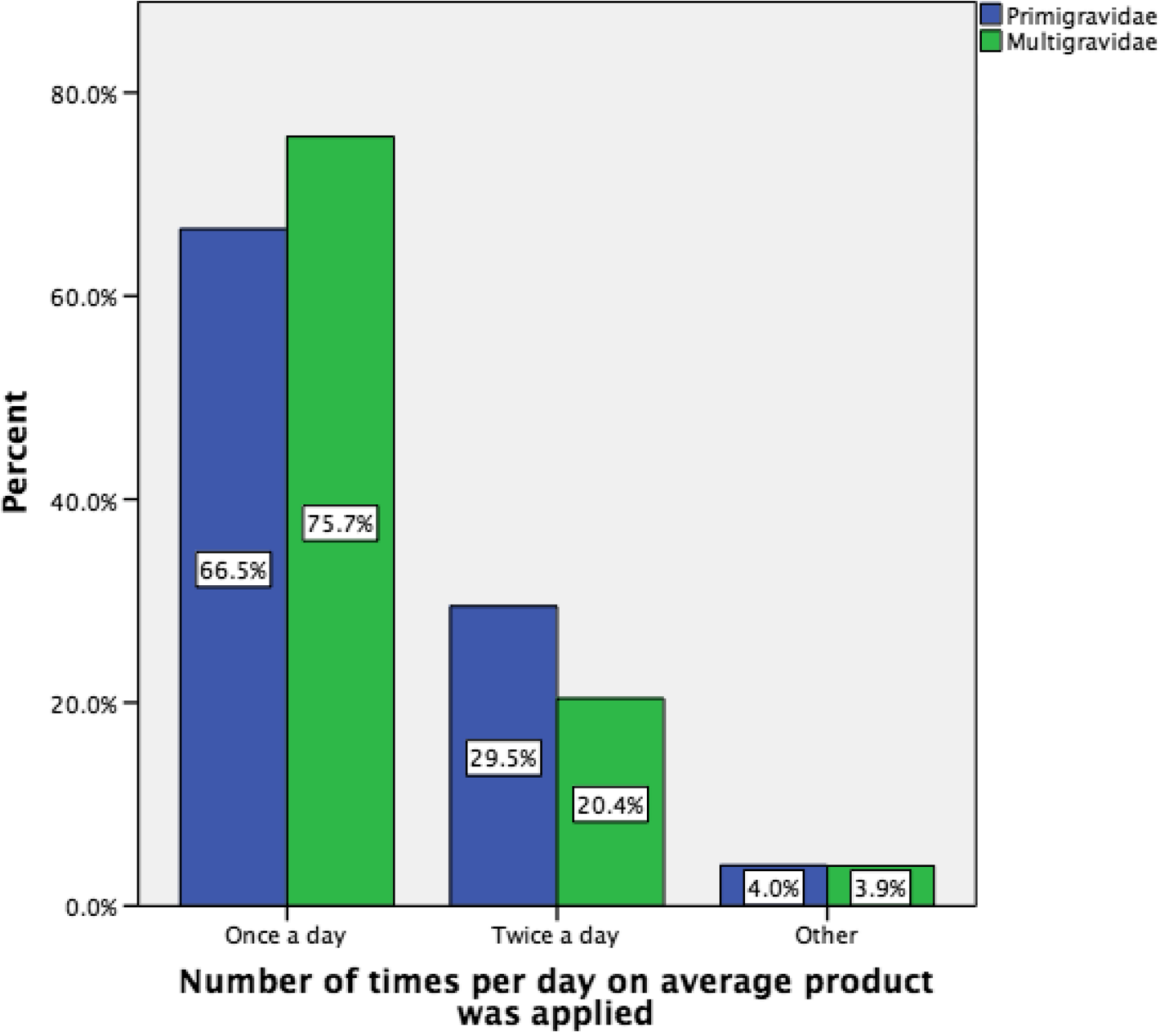
Number of times per day the product was applied by gravida

However, there was a statistical significant difference between the number of times per day the product was applied and being a multigravida or a primigravida woman (*p* < 0.05). The majority of mothers (primigravida and multigravida) used the product once or twice a day. However, more multigravida women used a product once a day in comparison with primigravida women (75.7 versus 66.5 % respectively, *p* = 0.018) and more primigravida used a product twice a day in comparison with multigravida (29.5 versus 20.4 % respectively, *p* = 0.014) indicating that primigravida women were applying the product more frequently during the day (Fig. 5).

**Fig. 6:**
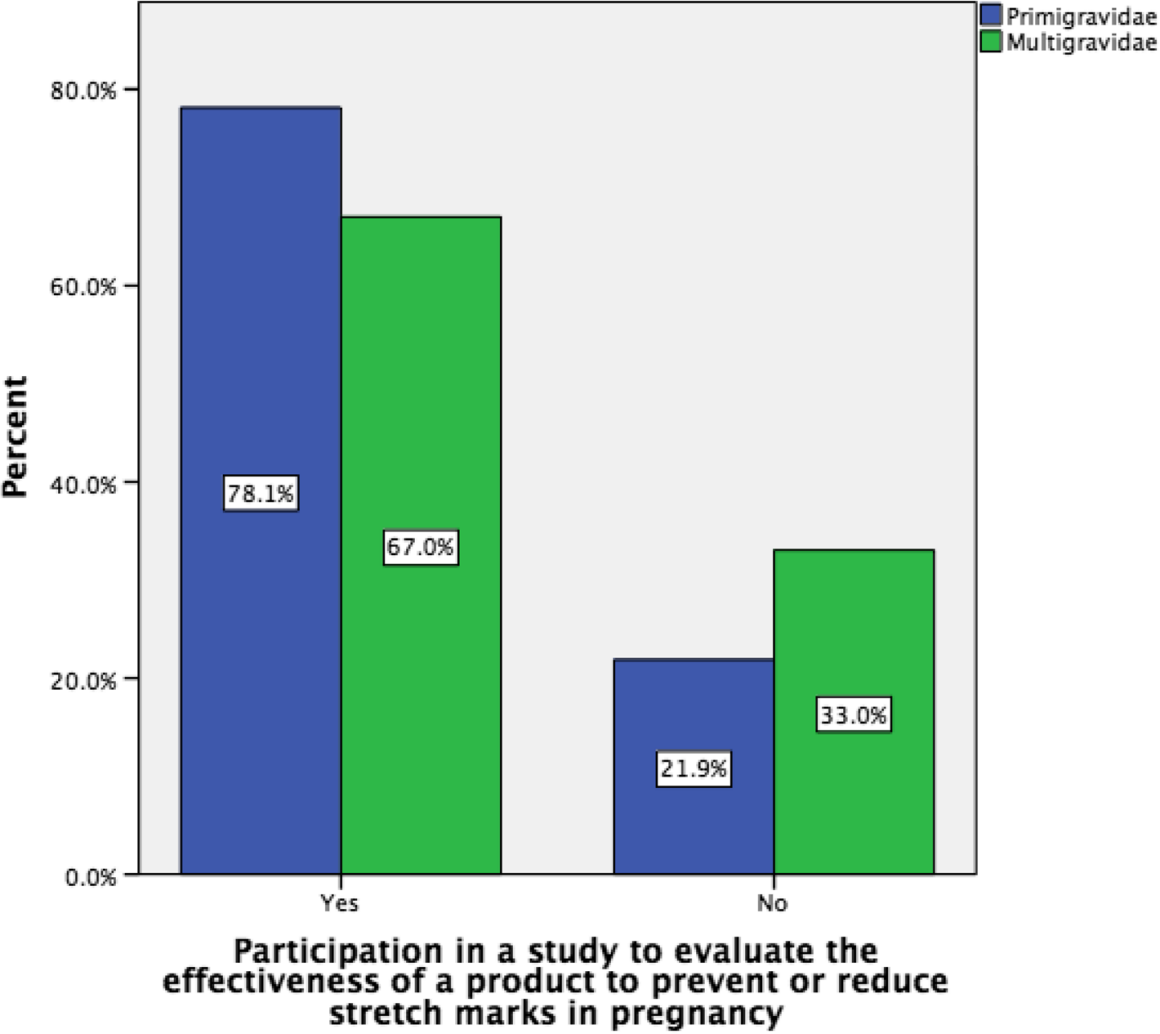
Consideration to participate in a future trial by gravida

The majority of respondents (75.5 %, *n* = 542) indicated that their decision to use a product to prevent stretch marks in pregnancy would be influenced by the findings of a research study and 68.3 % (*n* = 514) indicated that they would consider participating in a future trial of a product to prevent or reduce stretch marks in pregnancy. Further, significantly more primigravida women would consider participating in a future trial when compared with multigravida women (78.1 versus 67.0 % respectively, *p* = 0.001) (Fig. 6).

## Discussion

A large proportion of women in this survey used anti striae products, with 78.2 % of women indicating that they used one or more products to prevent or reduce the development of stretch marks during pregnancy. This is similar to a recent Japanese study [47] but higher than that reported by others [5, 26, 48]. Similar to the aforementioned Japanese study [47], we also found that significantly more primigravida women than multigravida women reported using a product to prevent stretch marks in pregnancy. Furthermore, primigravida were also more likely to spend more money and to apply the product more frequently compared to multigravida women. This suggests that primigravida women may be more motivated to attempt prevention or reduction in severity of striae. This is supported by our finding that primigravida women would be more likely to consider participating in a future trial compared to multigravida women.

A large range of products were used, as reported by others [4, 48]. Similarly, the use of more than one product has been identified by other researchers [5, 22, 48]. The most common product used by women in this study was Bio-oil, which consists of a plant and vitamin extract suspended in an oil base with fragrances and colouring added. We do not know how representative this is of other populations and occurs in the absence of high-quality evidence of the effectiveness of Bio-oil for the prevention of stretch marks in pregnancy, although it has been found to significantly improve stretch mark appearance in an exploratory study of non pregnant women [34]. Bio-oil is marketed widely in the print and electronic media and in recent years is more readily available in diverse high street locations, which may contribute to its popularity. One participant added how you 'hear lots about Bio-oil everywhere'.

Cocoa butter products were also used by a large proportion of women, as has been found by others [5] despite the lack of evidence to demonstrate their effectiveness in preventing striae gravidarum. One trial [3] that compared cocoa butter cream to a placebo found no significant difference between the control and intervention group. Similar results were found for cocoa butter lotion [49]. Cocoa butter is also present in some of the 'other products' used by women in this study and the effectiveness of these and the many other commercially available products used by women remains uncertain [35]. Some women may also have used cocoa butter lotion to prevent worsening of pre existing striae based on our finding of its use by women who developed stretch marks both prior to pregnancy and during the current pregnancy. In relation to olive oil, studies have yielded conflicting results. One early observational study [50] found that it did not prevent striae in primigravidae, but Davey [23] found in his non experimental study that olive oil massaged into the skin was associated with a lower incidence of stretch marks. More recently, olive oil was evaluated in two trials [51, 52] and neither supported its use for the prevention of striae gravidarum. In contrast to olive oil, bitter almond oil, has been found to be effective in a quasi-experimental study [53], which found that bitter almond oil and massage was effective, not the almond oil on its own. The use of baby oil has also been reported in other studies [5].

Women use various sources of information to help them to make pregnancy related decisions [54] and this is also true for anti striae products. Although other studies have found that women often seek advice from midwives and doctors on how to prevent striae [5, 22] this was not the case here. Advice from friends was the most commonly identified source of information. The role of friends, family and the internet has being identified by others [54]. Product advertisement was also influential in this study [48]. There is increasing awareness of the role of the internet to assist women in making decisions [55] and women are using the internet to inform pregnancy related choices [56, 57], especially in the early part of pregnancy [57]. While midwives have been identified as very important sources of information in pregnancy [54] this was not so in relation to anti stretch mark products but this is not surprising as many women had decided on which product to use in early pregnancy, before they had come into contact with a midwife or obstetrician.

It is also possible that the lack of consultation with health care professionals reflects the view that striae are a cosmetic or aesthetic concern [5, 17, 22, 30, 31] and therefore, unlike other physiological changes that arise in pregnancy, women might decide that they do not merit discussion with the maternity care provider. The majority of women using a product were applying it once a day, which concurs with advice to women participating in some studies [3, 49, 51, 58]. However, some advocate application at least twice a day [35].

Many of the products that women reported using have not been evaluated or, where they have been, have not been shown to prevent stretch marks. The majority of women in this survey indicated that they would be influenced by research evidence on the effectiveness of products for prevention or reduction of stretch marks in pregnancy and would be willing to participate in a trial of a product to prevent or reduce stretch marks in pregnancy. This is promising given the need for such evaluation and the recruitment problems for research studies generally and trials in particular [59].

The sample size and high response rate are key strengths of this study. Although the majority of the women were Irish, other ethnic groups were represented. Furthermore the sample closely represents the accessible [60] and national population [61] for 2014 in terms of women having their second or subsequent babies. They accounted for 59.8 % of all women in this study, which is very close to the accessible population (60.3 %) and the national picture (62 %).

More women developed stretch marks during the current pregnancy (46.7 %) than found in the Japanese cross sectional study [32] which included both primiparae and multiparae (39.1 %) but this was fewer than the 71.2 % of women who developed stretch marks found in a Polish study [28].

This survey also has some limitations including a non probability convenience sample that may not be truly representative of all pregnant women and how information provided by survey participants may be subject to recall bias [62]. While many women were still using the products, there may have been some bias with information recall, for example in relation to amount of money spent on products. Another possible consideration is that we could have asked women to identify specifically where they applied the anti striae product rather than asking a generic question on the application of a product to their skin to prevent stretch marks in pregnancy. Therefore, some caution is necessary when interpreting the findings.

## Conclusion

In conclusion, this is a large survey of women’s use of products to prevent or reduce the development of striae in pregnancy and highlights further the importance of preventing or minimising stretch marks to many women. However, there is a lack of high-quality evidence on the effectiveness of the products being used. This study, which is part of a planned investigation of topical products to prevent or reduce the development of stretch marks in pregnancy, follows on from the Cochrane Systematic Review exploring the effects of topical preparations on the prevention of stretch marks in pregnancy and provides the platform for a future trial to investigate the effectiveness of such products. It also provided us with an insight into the feasibility of recruiting women to a future trial. Future trials evaluating the effects of topical products on the prevention and reduction of stretch marks in pregnancy are necessary and can help to resolve the uncertainty around product efficacy and provide women with the information they need to make well-informed choices and to help health care professionals who are asked for advice by women.

## Abbreviations

HRQoL: Health related quality of life
